# Reliable machine learning deployment across domains in electrochemical energy storage materials

**DOI:** 10.1016/j.isci.2026.117160

**Published:** 2026-08-14

**Authors:** Henry R.N.B. Enninful, Muhammad Abdullah Khan

**Affiliations:** 1 Felix Bloch Institute for Solid State Physics, Faculty of Physics and Earth System Sciences, Leipzig University, Linnéstraße 5, 04103 Leipzig, Germany; 2 Renewable Energy Advancement Laboratory (REAL), Department of Environmental Sciences, Quaid-i-Azam University Islamabad, Islamabad 45320, Pakistan

**Keywords:** energy storage materials, machine-learning reliability, transferability, epistemic uncertainty, domain shift, latent representations, simulation-to-experiment transfer, materials discovery

## Abstract

Machine learning accelerates the discovery of electrochemical energy-storage materials but can fail under domain shift. We audit a fixed pretrained latent representation for porous supercapacitor materials, spanning porous carbons, metal-organic frameworks (MOFs), simulated MOFs, and external carbon/covalent triazine framework (CTF) datasets. Deployment risk is governed by latent geometry and local representational support rather than domain labels alone. Nearest-neighbor target-property discrepancy increases in extrapolative regions, while sparse neighborhoods show higher epistemic uncertainty. Combining latent distance with uncertainty yields interpretable safety maps that flag supported, cautionary, and unsupported deployment regimes, providing a reliability-first safeguard for machine learning (ML)-guided materials screening.

## Highlights

• Latent geometry governs transfer reliability in porous materials
• Sparse neighborhoods increase uncertainty and deployment risk
• Target-property discontinuities expose unsafe transfer regimes
• Safety maps flag predictions for rejection or cautious use

## Graphical abstract

**Figure:**
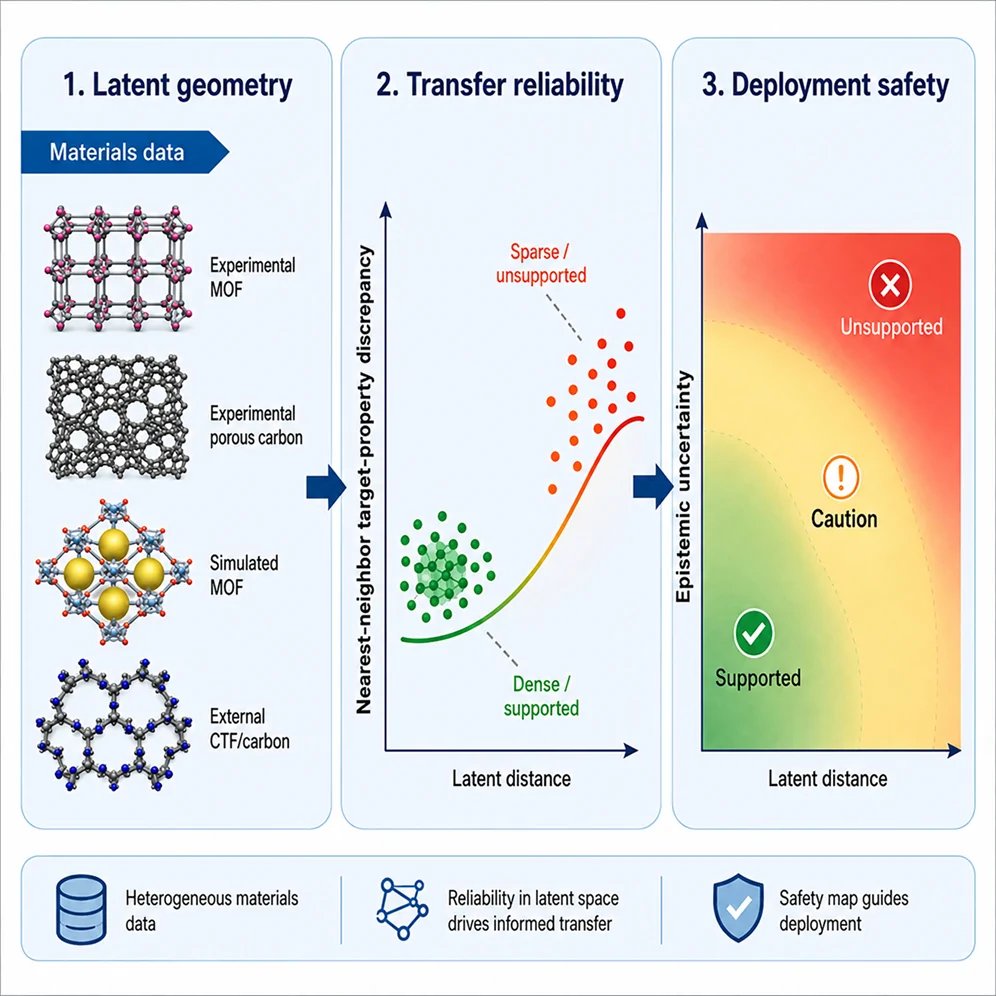

## Teaser

Machine learning; Electrochemical energy storage; Materials science

## Introduction

The urgent need for sustainable energy technologies has driven rapid interest in data-driven methods to accelerate materials discovery for energy storage and conversion applications.^1^ Machine learning (ML) models are now routinely used to predict properties of batteries, supercapacitors, porous electrodes, and electrochemical materials, offering the promise of reducing experimental cost and time-to-discovery through high-throughput screening and surrogate modeling.^2^^,^^3^^,^^4^^,^^5^ In particular, porous materials such as porous carbons, metal-organic frameworks (MOFs), and covalent triazine frameworks (CTFs) have emerged as attractive candidates for next-generation energy storage due to their tunable chemistry, high surface area, and structural diversity.^6^^,^^7^^,^^8^^,^^9^^,^^10^

Despite these advances, a growing gap has emerged between model performance under conventional validation settings and reliability under real deployment conditions. Most ML models for materials science are trained and evaluated assuming that training and test data are drawn from the same distribution, an assumption that is rarely satisfied in practice.^11^^,^^12^ In energy research, models are often transferred across material classes, between simulated and experimental datasets, or to newly synthesized compounds that lie outside the domain of available data.^13^^,^^14^^,^^15^ Under these conditions, even models with excellent cross-validation accuracy may fail silently, producing predictions that appear confident but are physically unreliable.^16^

This challenge is particularly acute for energy storage materials, where experimental validation is costly and misdirected predictions can significantly slow progress. For example, models trained on simulated adsorption or electrochemical data are often applied directly to experimental systems, despite systematic discrepancies arising from defects, synthesis variability, and measurement noise.^17^^,^^18^ Similarly, ML models trained on one class of porous materials, such as porous carbons or MOFs, are increasingly used to guide exploration in emerging material families such as COFs and CTFs, where structural motifs and structure-property relationships may differ substantially.^19^^,^^20^ Quantifying when such transfers are reliable, and when they are not, remains an open and underexplored problem.

Recent work has highlighted the importance of uncertainty quantification and out-of-distribution (OOD) detection as tools for addressing these limitations.^21^^,^^22^^,^^23^^,^^24^^,^^25^ Bayesian neural networks, ensemble methods, and Monte Carlo dropout have been proposed to estimate predictive uncertainty in materials models.^26^^,^^27^^,^^28^ However, uncertainty estimates alone do not fully resolve the question of why and where models fail under domain shift, nor do they provide actionable criteria for deciding when predictions should be trusted, flagged for caution, or rejected entirely.^29^ Moreover, uncertainty metrics are often reported without reference to the underlying representation geometry learned by the model, obscuring the connection between data coverage, similarity, and transferability.

Latent representations learned by neural networks provide a natural framework for addressing these challenges. Recent representation-learning work for cross-domain materials datasets has shown that shared latent spaces can be audited for domain alignment, neighborhood structure, and transferability across heterogeneous materials domains.^30^ Prior studies have shown that distances and densities in latent space correlate with prediction error, uncertainty, and extrapolation risk across a range of scientific domains.^22^^,^^31^^,^^32^^,^^33^ In materials science, latent-space analyses have been used to visualize chemical similarity, identify data gaps, and guide active learning strategies.^34^^,^^35^ However, systematic audits of domain-pair transferability—examining how reliability depends on both the source and target domains—are limited, particularly in the context of energy storage materials.

Here, we present a transferability and safety audit of a fixed pretrained latent representation generated during an earlier stage of our cross-domain materials representation-learning workflow.^30^ The encoder is treated as a frozen deployment object and applied to porous supercapacitor materials spanning porous carbons, MOFs, and CTFs across experimental and simulated datasets. Using this shared latent encoder, we analyze representation geometry, neighborhood structure, and local density to quantify how deployment-risk indicators and epistemic uncertainty evolve with latent distance. Domain-pair-specific transfer diagnostics reveal strong asymmetries in reliability that are obscured by aggregate performance metrics, exposing where apparent accuracy may mask unsupported or extrapolative deployment regimes.

By integrating geometric and uncertainty signals, we introduce uncertainty-aware safety maps, as interpretable deployment-risk diagnostics under domain shift. Exploratory calibration and risk-coverage analyses are discussed in the Supplementary Information, but the main conclusions rely on directly interpretable non-parametric diagnostics: latent distance, local density, epistemic uncertainty, and nearest-neighbor target-property discrepancy. This reliability-first framework moves beyond implicit assumptions of transferability, enabling informed use of ML predictions and helping ensure that ML-guided screening accelerates—rather than misdirects—experimental effort. Although demonstrated for electrochemical energy storage materials, the challenges addressed here (i.e., transferability, extrapolation, and uncertainty under domain shift) are pervasive across data-driven energy and materials research. As ML models are increasingly deployed in catalysis, electrochemical conversion, and energy systems design, the ability to identify when predictions should not be trusted becomes as important as predictive accuracy itself.

Rather than improving predictive accuracy, this work addresses a complementary and underexplored problem: how to determine when ML predictions should be flagged for cautious interpretation or rejection in materials discovery workflows. Existing OOD detection and uncertainty quantification methods typically provide scalar indicators of predictive confidence but do not explicitly resolve why models fail under domain shift or how reliability varies across domain pairs. In contrast, the present work introduces a geometry-resolved audit framework that links high-risk deployment regimes to latent neighborhood structure and representational support. It also reveals asymmetric transferability across domain pairs and provides actionable deployment criteria, such as safety maps and rejection rules, without requiring model retraining.

## Results

### Latent geometry and epistemic uncertainty jointly define supported and high-risk regions of materials space

**Figure 1:**
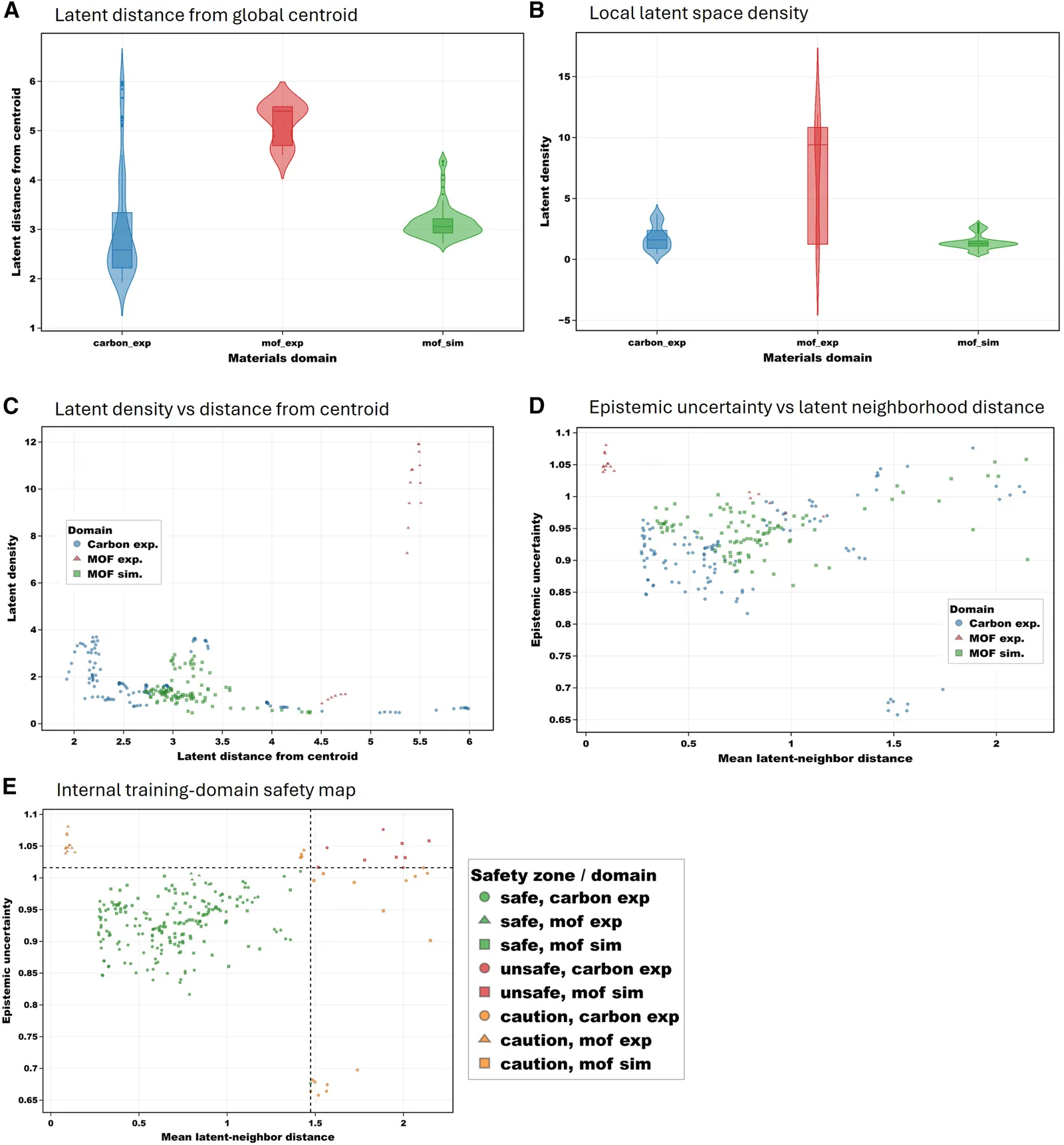
Latent geometry and epistemic uncertainty structure the reliability of materials representations (A) Distribution of latent radius (distance to the global centroid) across materials domains. (B) Distribution of local latent density estimated from k-nearest neighbors, highlighting domain-dependent representational support. (C) Latent density versus latent radius, showing that sparsity and distance are not strictly coupled. (D) Epistemic uncertainty estimated *via* Monte Carlo dropout as a function of latent distance. (E) Internal training-domain safety map combining latent distance and epistemic uncertainty for the datasets used in the latent-space audit. Thresholds are defined from the training-domain distribution and classify samples into safe, caution, and unsafe regions.

Dataset composition and target availability are summarized in Table 1 because latent-space reliability depends on domain size and sampling density. Figures 1A–1E characterize the learned representation and its deployment-risk structure.

**Table 1:** Dataset composition used for latent-space reliability analysis

Domain label	Materials class	Data origin	Role in analysis	No. of samples	Available targets
carbon_exp	Porous carbons	Experimental	Training-domain audit	*n* = 122	*C*_*g*_, *C*_*v*_
mof_exp	MOFs	Experimental	Training-domain audit	*n* = 18	*C*_*g*_, *C*_*v*_
mof_sim	MOFs	Simulated	Training-domain audit	*n* = 102	*C*_*g*_, *C*_*v*_
extra_carbon_exp	Porous carbons	Experimental	Inference-only external test	*n* = 37	geometry-only
extra_CTF_exp	CTFs	Experimental	Inference-only external test	*n* = 6	geometry-only

We first examine latent radius, defined as distance from the global latent centroid (Figure 1A). Experimental porous carbons span a broad radial range, simulated MOFs occupy a compact intermediate regime, and experimental MOFs are displaced toward larger radii. Local density, defined as the inverse mean distance to nearest neighbors, further shows that carbon materials and simulated MOFs are relatively compact, whereas experimental MOFs are more heterogeneous (Figure 1B). The joint density-radius view (Figure 1C) shows that global distance and local support are not equivalent: some distant MOFs remain locally dense, while some more central carbon or simulated MOF samples are of lower density. Thus, distance alone is insufficient to define reliability.

We next relate latent geometry to epistemic uncertainty estimated by Monte Carlo dropout. Uncertainty generally increases in sparse or peripheral regions, but the trend is not strictly monotonic (Figure 1D). Some experimental MOFs show high uncertainty despite short mean latent-neighbor distances, whereas a small subset of distant carbon samples retains low uncertainty. Latent distance and epistemic uncertainty therefore provide complementary information.

Combining latent distance and epistemic uncertainty yields an internal safety map for the training-domain datasets (Figure 1E). Percentile-based thresholds partition samples into interpolation-like, cautionary, and high-risk regions. Most porous carbon and simulated MOF samples lie in the interpolation-like region, whereas experimental MOFs are enriched in cautionary regimes, establishing the internal reliability landscape used for subsequent deployment analyses.

Together, these results show that latent geometry and epistemic uncertainty define well-supported, cautionary, and high-risk regimes that motivate the following transferability and deployment-risk analyses in this work.

### Latent geometry and epistemic uncertainty define reliability across materials domains

Because both gravimetric and volumetric capacitance labels are available, all transferability and reliability analyses reported in this section are target-resolved unless otherwise noted. We examine how latent geometry, neighborhood structure, and epistemic uncertainty jointly govern deployment-risk behavior across heterogeneous materials domains.

**Figure 2:**
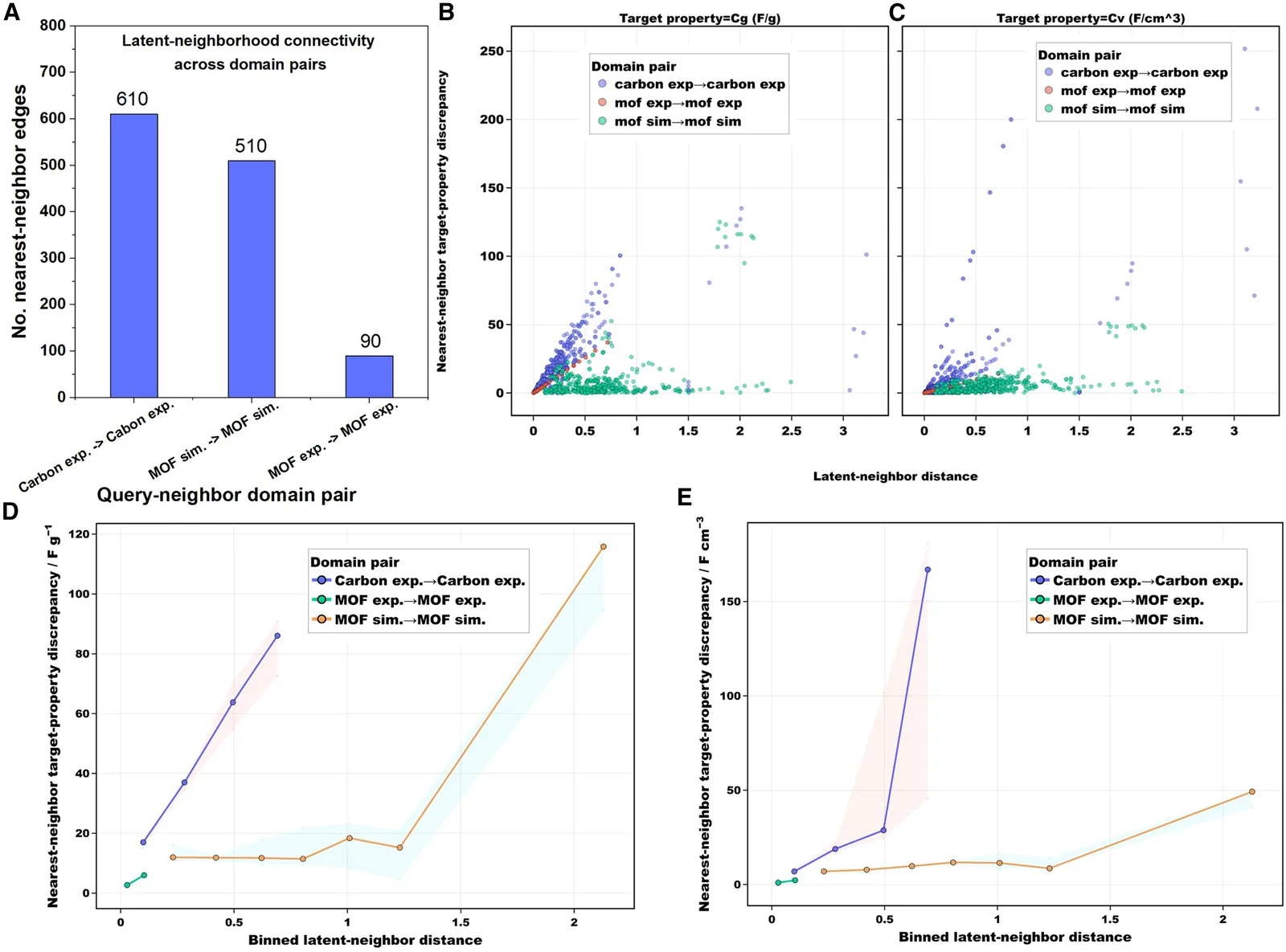
Latent connectivity, extrapolation distance, and uncertainty jointly govern transfer reliability and deployment safety (A) Latent neighborhood connectivity across domain pairs quantified by k-nearest-neighbor edge counts, revealing large differences in representational support. (B) Absolute nearest-neighbor target-property discrepancy as a function of latent distance for gravimetric capacitance, *C*_*g*_, showing increased local target-property discontinuity at larger latent-neighbor distances rather than direct supervised prediction error. (C) Absolute nearest-neighbor target-property discrepancy as a function of latent distance for volumetric capacitance, *C*_*v*_, showing increased local target-property discontinuity at larger latent-neighbor distances rather than direct supervised prediction error. (D) Distance-binned nearest-neighbor target-property discrepancy envelopes for gravimetric capacitance, *C*_*g*_, showing the median, interquartile range, and 90th-percentile discrepancy and highlighting domain-dependent reliability limits. (E) Distance-binned nearest-neighbor target-property discrepancy envelopes for volumetric capacitance, *C*_*v*_, showing the median, interquartile range, and 90th-percentile discrepancy and highlighting domain-dependent reliability limits.

We first quantify latent neighborhood structure using k-nearest-neighbor (kNN) connectivity within the learned representation (Figure 2A). Experimentally measured porous carbon materials exhibit the highest within-domain connectivity, followed by simulated MOFs, whereas experimentally measured MOFs display substantially fewer kNN edges. This reduced connectivity indicates weaker local representational support for experimental MOFs, consistent with their more fragmented latent neighborhood structure in the learned latent space.

The consequences of this latent structure are illustrated using nearest-neighbor target-property discrepancy, plotted as a function of latent distance (Figure 2B). Short-distance neighbor pairs generally show low target-property discrepancy across domains and both targets, whereas discrepancy increases for more distant neighbor pairs. Across the analyzed domains, regions of larger latent distance and reduced local support coincide with increased nearest-neighbor target-property discrepancy and elevated uncertainty (Figures 2B–2E and S1), demonstrating that geometry-based indicators provide conservative diagnostics of deployment risk rather than direct measures of supervised prediction error. Complementarily, density-based diagnostics show that large nearest-neighbor target-property discrepancies, rather than direct prediction errors, concentrate in low-density regions of latent space across both targets, confirming that local representational support is a primary determinant of transfer reliability (Figure S2A). Latent density provides information complementary to latent distance because it directly reflects the amount of local representational support available for interpolation, whereas distance alone cannot distinguish between well-supported peripheral regions and truly isolated extrapolative regimes.

To quantify distance-dependent target-property discontinuity systematically, we construct distance-binned nearest-neighbor target-property discrepancy envelopes for gravimetric and volumetric capacitance, respectively (Figures 2D and 2E). For simulated MOFs, median discrepancy remains low and interquartile ranges remain narrow across distance bins, indicating stable local structure–property consistency. In contrast, experimental carbon and experimental MOF domain pairs show stronger increases in discrepancy and/or envelope width at larger latent-neighbor distances, indicating that latent separation is associated with reduced local target-property smoothness. A relaxed-parameter sensitivity analysis confirms that these envelope trends and the resulting domain ordering are robust to reasonable choices of neighborhood size and binning thresholds (Figure S1).

**Figure 3:**
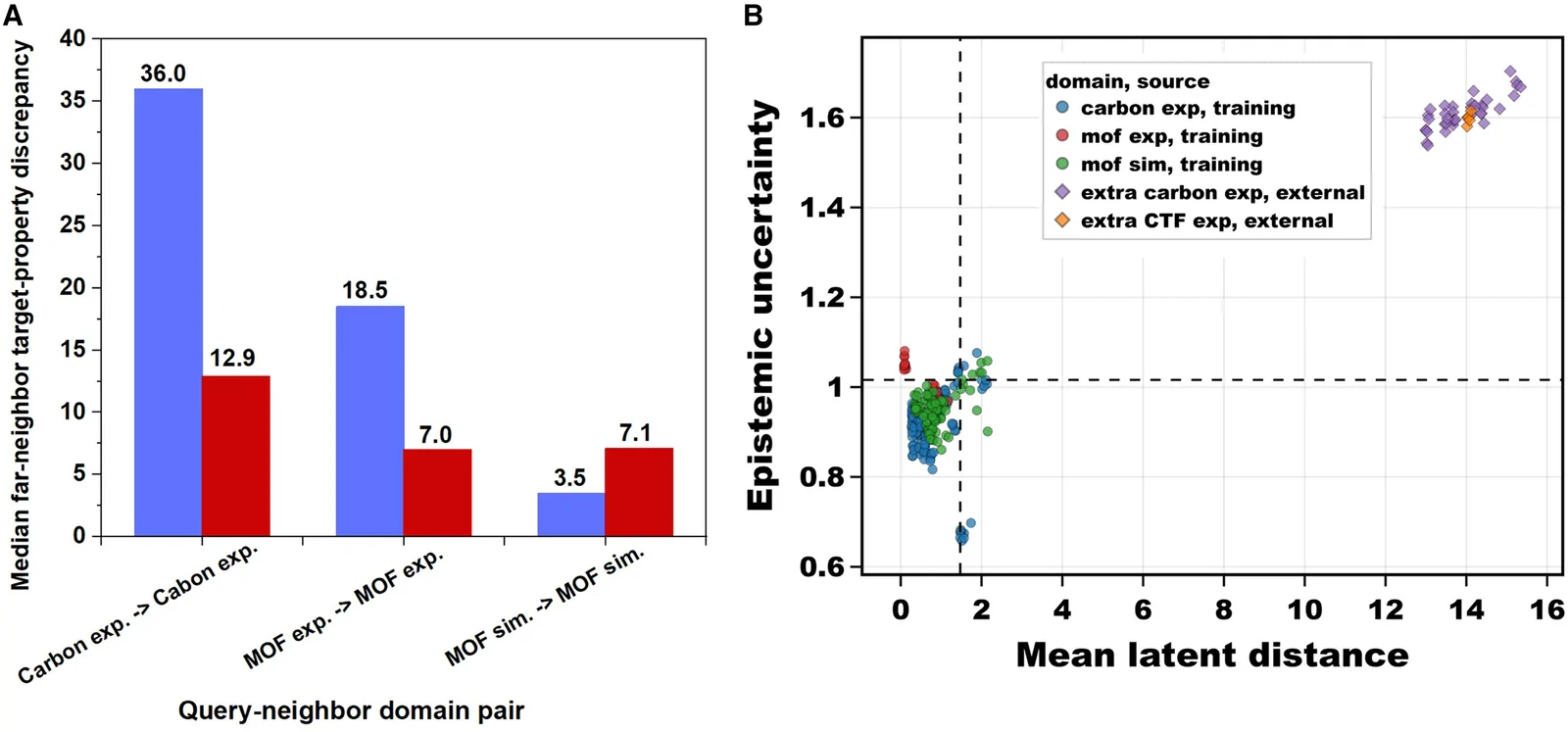
Domain-pair reliability and external deployment maps identify supported and high-risk transfer regimes (A) Median far-neighbor nearest-neighbor target-property discrepancy summarizing domain-pair reliability for both targets. (B) External deployment/OOD map applying the training-domain distance-uncertainty thresholds to inference-only experimental datasets, including additional porous carbons and CTFs. Training-domain samples are shown as the reference distribution, while external samples indicate deployment regimes with reduced latent support. Related density- and uncertainty-based diagnostics, along with sensitivity analyses of the nearest-neighbor target-property discrepancy envelopes, are provided in Figures S1 and S2.

Domain-pair reliability is summarized by the median far-neighbor nearest-neighbor target-property discrepancy (Figure 3A). Simulated MOFs exhibit the lowest far-neighbor discrepancy for both capacitance targets, followed by experimental MOFs, while carbon experimental materials show the largest far-neighbor discrepancy despite constituting the largest fraction of the training data. This ordering mirrors latent connectivity rather than dataset size or provenance, underscoring that representational support—not domain identity—governs transfer reliability.

We then apply the same distance-uncertainty criteria to external inference-only datasets to evaluate deployment risk (Figure 3B). In contrast, the internal safety map in Figures 1E and 3B includes additional experimental porous carbon and CTF samples introduced only at inference time for the latter figure. These external samples occupy regions of increased latent distance and/or elevated epistemic uncertainty relative to the training-domain distribution, indicating reduced representational support. Their placement outside the well-supported internal regime shows how the audit can flag deployment cases that should be treated cautiously or rejected under conservative screening rules. Independent neighborhood-based uncertainty proxies further show that uncertainty increases with latent extrapolation (latent radius) and decreases with latent density across both targets (Figures S2B and S2C), supporting the interpretation that these external samples lie in strongly unsupported representational regimes where predictions should be rejected or treated as unsupported under conservative deployment rules. An internal leave-one-out latent-neighborhood validation further supports the association between the proposed reliability indicators and predictive degradation for samples with available *C*_*g*_ and *C*_*v*_ labels (Figure S3).

These results establish a unified picture in which latent connectivity governs interpolation capacity, latent distance is associated with increased nearest-neighbor target-property discrepancy, and epistemic uncertainty identifies deployment risk. Supplementary analyses (Figures S1 and S2) reinforce that these reliability trends are consistent across targets and robust to analysis hyperparameters, enabling a geometry-aware, domain-agnostic framework for safe ML deployment in electrochemical energy storage materials. Accordingly, our conclusions do not depend on conservative filtering choices but instead reflect intrinsic features of the learned latent geometry.

### Case studies of reliable versus unsafe deployment

To illustrate how the proposed reliability audit operates at the level of individual samples, we examined representative cases selected from distinct regions of the latent reliability map. These examples show how latent distance, local density, epistemic uncertainty, and nearest-neighbor target-property discrepancy provide complementary information about deployment risk.

#### Case study A: Interpolation-like regimes in dense latent regions

Samples located in high-density, low-distance regions of latent space exhibit low nearest-neighbor target-property discrepancy and low epistemic uncertainty. Representative porous carbon samples in this regime show dense kNN connectivity and minimal variation in capacitance among latent neighbors. These samples lie within well-supported regions of the training distribution and are, therefore, consistent with interpolation-like behavior. In this regime, latent similarity preserves local target-property similarity, indicating that dense representational support enables comparatively reliable predictions.

#### Case study B: Silent-failure risk despite moderate uncertainty

In contrast, some samples exhibit large nearest-neighbor target-property discrepancies even when epistemic uncertainty is not extreme. Such cases are important because uncertainty alone may not fully capture local discontinuities in target behavior. In these regions, latent neighbors are geometrically close but differ substantially in capacitance, indicating that the learned representation does not locally preserve the relevant structure-property relationship. These cases, therefore, represent a silent-failure risk: predictions may appear acceptable from uncertainty alone but should be treated cautiously when geometric and target-discrepancy diagnostics are considered jointly.

#### Case study C: Sparse extrapolative regimes

Samples located at large latent distances and low local density occupy poorly supported regions of representation space. These sparse neighborhoods are associated with large nearest-neighbor target-property discrepancies, indicating that the model is operating outside regions where local interpolation can be considered reliable. Such samples should, therefore, be flagged for cautious interpretation or rejected under conservative deployment rules. This regime highlights that high latent distance combined with low density provides a strong indication of extrapolative deployment risk.

To summarize these regimes quantitatively, Table S1 (supplemental information) reports representative cases selected from the latent-neighborhood diagnostics for gravimetric and volumetric capacitance. The reported nearest-neighbor target-property differences quantify local target discontinuity in latent space and are interpreted as representation-smoothness diagnostics rather than direct supervised prediction errors. Case A corresponds to dense, low-distance interpolation with small local target discontinuity, whereas cases B and C illustrate elevated deployment risk arising from local target discontinuity or sparse latent support. To visualize these regimes, the representative Table S1 cases were projected onto the latent distance-epistemic uncertainty safety map (Figure S4), showing how interpolation-like, silent-failure-risk, and sparse extrapolative samples occupy distinct regions of the deployment-risk space.

Representative cases were selected separately for *C*_*g*_ and *C*_*v*_. Nearest-neighbor target-property differences quantify local target discontinuity between latent neighbors and are interpreted as representation-smoothness diagnostics rather than direct supervised prediction errors. No sample satisfying the combined criteria for a locally supported peripheral regime was identified under the present thresholds.

## Discussion

This work shifts the focus from maximizing predictive accuracy to identifying when predictions should be accepted, treated cautiously, or considered unsupported under domain shift. ML models increasingly guide synthesis, electrochemical characterization, and theoretical follow-up in electrochemical energy storage research, yet they are often deployed beyond their training distributions while being evaluated mainly by aggregate in-distribution metrics. Our results show that strong apparent performance can co-exist with systematic failure when models are transferred across material classes or extrapolated in representation space.

A central contribution is to frame deployment reliability as a property of learned representation geometry rather than domain labels, data provenance, or dataset size alone. A read-only audit of a pre-trained encoder shows that neighborhood connectivity, local density, nearest-neighbor target-property discrepancy, and epistemic uncertainty jointly define deployment risk. Well-supported regimes are dense and locally smooth; sparse or extrapolative regimes show larger discrepancies and higher uncertainty. The framework, therefore, extends conventional OOD detection by resolving domain-pair transfer behavior and translating latent geometry into safety maps without retraining.

Latent distance alone is insufficient because it cannot distinguish peripheral but locally supported samples from isolated regimes where interpolation breaks down. Local density provides the missing measure of representational support. This explains why some distant samples remain locally coherent, whereas others with comparable distances become unreliable because their neighborhoods are sparse or fragmented.

Domain-pair analyses reveal asymmetries hidden by standard validation. Some experimental domains have fragmented neighborhoods and large far-neighbor discrepancies despite larger sample size, whereas coherent simulated domains support stable interpolation across distance bins. Thus, transfer reliability follows latent connectivity rather than whether a dataset is experimental, simulated, large, or small.

Safety maps combining latent distance and epistemic uncertainty identify unsupported regions without requiring labels for new samples. Applied to external experimental datasets, these maps flag samples with reduced representational support that should be treated cautiously rather than automatically trusted. Neighborhood-based diagnostics and leave-one-out validation support the association between sparse support, higher uncertainty, and predictive degradation for samples with available *C*_*g*_ and *C*_*v*_ labels.

**Figure 4:**
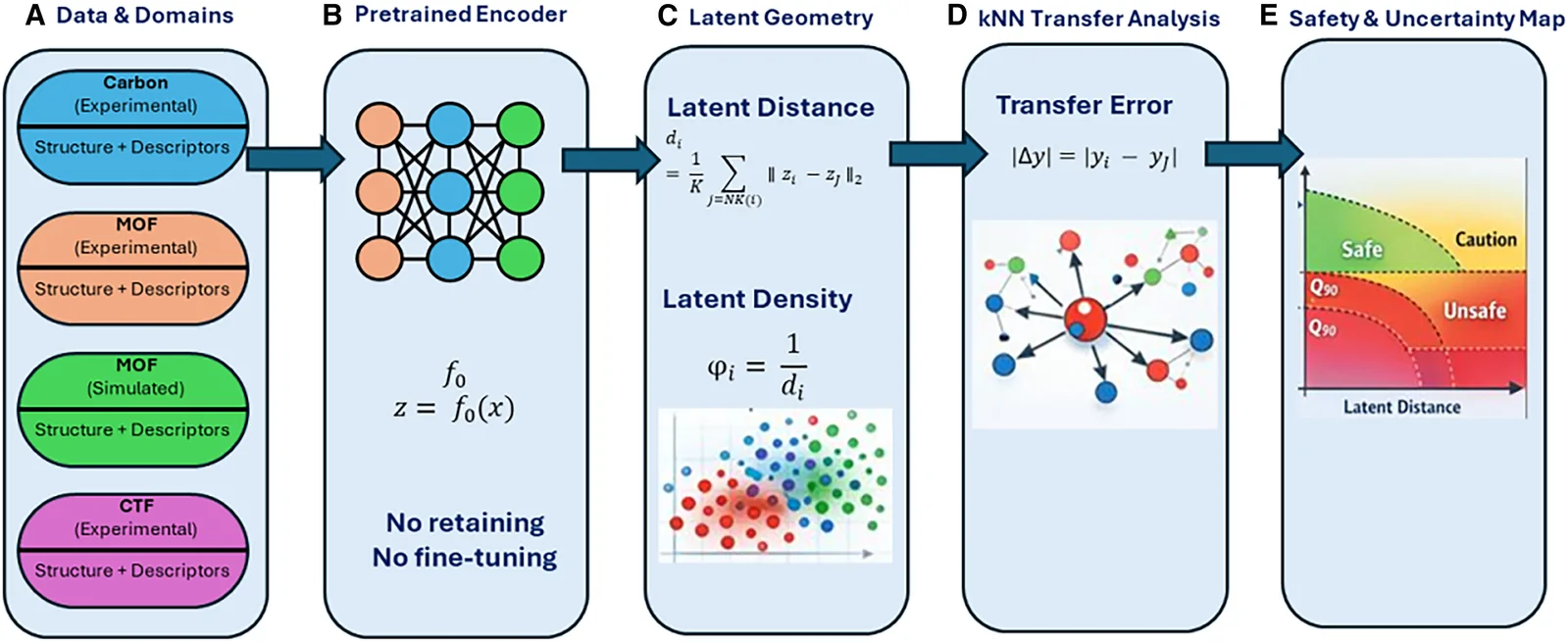
Schematic overview of the latent transferability and uncertainty audit (A) Experimental and simulated materials datasets from multiple domains are mapped to a shared feature representation. (B) A pre-trained neural encoder is used in inference mode to generate latent representations without retraining. (C) Latent geometry is quantified using neighborhood-based distance and density metrics. (D) k-nearest-neighbor graphs define cross-domain transfer relationships, enabling local estimation of nearest-neighbor target-property discrepancy between related materials. (E) Latent distance and epistemic uncertainty are combined to construct a safety-aware deployment map, identifying regions of reliable transfer, caution, and out-of-distribution behavior for new materials.

Sensitivity analyses show that these reliability trends are robust to reasonable neighborhood sizes, binning thresholds, and filtering parameters. Their consistency across both capacitance targets suggests that the framework captures general features of representational support rather than target-specific fluctuations. A schematic overview of the latent transferability and uncertainty audit workflow is shown in Figure 4 (STAR Methods).

Practically, the work supports selective deployment: knowing when predictions should not be trusted is as important as achieving high accuracy in well-supported regimes. Because the diagnostics operate on a pre-trained representation and do not require labels for new samples, they can be added to existing screening workflows as safeguards against high-risk extrapolation. The same principle should extend to other materials-learning settings with meaningful latent spaces and comparable descriptors.

Here, “reliable” means that a prediction lies in a region of latent space with high local density, low epistemic uncertainty, and bounded nearest-neighbor target-property discrepancy. It does not guarantee predictive accuracy without independent validation. The framework, therefore, identifies supported and unsupported deployment regimes and complements, but does not replace experimental testing or external supervised validation.

### Limitations of the study

This study audits one fixed pretrained latent encoder generated during an earlier representation-learning workflow. The encoder is treated as a frozen deployment object rather than a newly optimized or universally optimal architecture. Alternative encoders, regularization settings, or retrained models may produce different latent geometries and safety boundaries.

Second, nearest-neighbor target-property discrepancy is a local representation-smoothness diagnostic, not a substitute for prospective experimental validation or supervised prediction error on entirely unseen materials. The framework is most useful for identifying regions where latent proximity fails to preserve target-property similarity.

Third, the study is limited to experimental porous carbons, experimental and simulated MOFs, external porous carbon/CTF datasets, and gravimetric and volumetric capacitance. Broader validation across additional materials classes, electrolytes, device configurations, and targets is needed. The empirical safety thresholds may also require re-calibration for other datasets, models, or screening objectives.

Finally, the framework depends on a meaningful latent representation and harmonized descriptors. If key physical descriptors are missing or the representation does not preserve structure-property relationships, the diagnostics may be less informative. Future work should test the approach prospectively, compare multiple encoder variants, and integrate physics-informed descriptors or uncertainty-calibrated predictors.

## Resource availability

### Lead contact

Requests for further information and resources should be directed to and will be fulfilled by the lead contact, Henry R.N.B. Enninful (hrnbenninful@gmail.com).

### Materials availability

No materials were used or generated in this study.

### Data and code availability

• The curated datasets, input arrays, target arrays, feature list, feature scaler, fixed pretrained encoder, preprocessing artifacts, reproducibility files, generated outputs, and analysis artifacts are publicly available in *Zenodo* under accession code https://doi.org/10.5281/zenodo.20448825.^36^ All data reported in this paper will be shared by the lead contact upon request.
• The custom inference-only reliability-audit code required to reproduce the latent-distance and local-density analyses, kNN/domain-pair diagnostics, Monte Carlo dropout uncertainty estimates, nearest-neighbor target-property discrepancy analyses, safety maps, sensitivity analyses, internal leave-one-out validation, figures, and supplementary outputs is publicly available in *Zenodo* under accession code https://doi.org/10.5281/zenodo.20448825.^36^ The development code repository is available at GitHub under accession https://github.com/HRNBEnninful/Latent_Space_Transfer. No access restrictions apply.
• The present work audits one fixed pretrained latent representation and its associated preprocessing pipeline. Therefore, the reported safety boundaries, latent-neighborhood diagnostics, and deployment-risk classifications are specific to the archived encoder, scaler, feature list, and analysis artifacts used in this study rather than universal boundaries for all representation-learning models. Any additional information required to re-analyze the data reported in this paper is available from the lead contact upon request.

## Acknowledgments

The work leading to this publication was supported by the PRIME program of the 10.13039/100021828German Academic Exchange Service (10.13039/501100001655DAAD) with funds from the 10.13039/501100002347German Federal Ministry of Education and Research (10.13039/501100002347BMBF – Project ID 57701188).

## Author contributions

H.R.N.B.E., conceptualization, data curation, formal analysis, funding acquisition, investigation, methodology, project administration, resources, software, supervision, visualization, and writing – original draft and writing – review and editing; M.A.K, conceptualization, visualization, validation, supervision, and writing – review and editing.

## Declaration of interests

The authors declare no competing interests.

## Declaration of generative AI and AI-assisted technologies in the writing process

During preparation of this manuscript, the authors used ChatGPT version 5.5 (OpenAI) for language polishing and readability improvement only. The authors reviewed and edited all AI-assisted text and take full responsibility for the final content. No generative AI or AI-assisted tools were used to create scientific figures, graphical abstracts, schemes, or artwork.

## STAR★Methods

### Key resources table

**Table:** 

REAGENT or RESOURCE	SOURCE	IDENTIFIER
**Deposited data**		

Curated experimental porous carbon dataset	Cui et al.^37^; Borchardt et al.^38^; Pal et al.^39^; Liu et al.^40^; Hurilechaoketu et al.^41^; Liu et al.^42^; Yang et al.^43^; Chen et al.^44^; Zhao et al.^45^; Sillars et al.^46^; archived in this study	Zenodo accession code: https://doi.org/10.5281/zenodo.20448825.^36^; reported as carbon_exp
Experimental metal–organic framework dataset	Gittins et al.^47^; archived in this study	Zenodo accession code: https://doi.org/10.5281/zenodo.20448825.^36^; reported as mof_exp
Simulated metal–organic framework dataset	Wang et al.^48^; archived in this study	Zenodo accession code: https://doi.org/10.5281/zenodo.20448825.^36^; reported as mof_sim
Additional external experimental porous carbon dataset	Yan et al.^49^; Yan et al.^50^; archived in this study	Zenodo accession code: https://doi.org/10.5281/zenodo.20448825.^36^; reported as extra_carbon_exp
External experimental covalent triazine framework (CTF) dataset	Hao et al.^9^; archived in this study	Zenodo accession code: https://doi.org/10.5281/zenodo.20448825.^36^; reported as extra_CTF_exp

**Software and algorithms**		

Custom inference-only reliability-audit code	This study	Zenodo accession code: https://doi.org/10.5281/zenodo.20448825.^36^; custom inference-only reliability-audit code
Development code repository	This study	GitHub repository accession: HRNBEnninful/Latent_Space_Transfer; https://github.com/HRNBEnninful/Latent_Space_Transfer
Python version 3.13.3	Python Software Foundation	https://www.python.org/
PyTorch version 2.11.0	PyTorch Foundation	https://pytorch.org/
scikit-learn version 1.8.0	scikit-learn developers	https://scikit-learn.org/
NumPy version 2.4.4	NumPy developers	https://numpy.org/
pandas version 3.0.2	pandas development team	https://pandas.pydata.org/
SciPy version 1.17.1	SciPy developers	https://scipy.org/
Plotly version 6.7.0	Plotly Technologies Inc.	https://plotly.com/python/
UMAP-learn version 0.5.12	McInnes et al.^51^	https://umap-learn.readthedocs.io/
Pre-trained latent encoder	Enninful et al.^30^; used as a frozen model in this study	Zenodo accession code: https://doi.org/10.5281/zenodo.20448825.^36^; fixed pretrained latent encoder archived with reproducibility package
Feature scaler, feature list, input arrays, and target arrays	This study	Zenodo accession code: https://doi.org/10.5281/zenodo.20448825.^36^; feature scaler, feature list, input arrays, and target arrays archived with reproducibility package

### Experimental model and study participant details

This study did not involve experimental models, biological materials, human participants, animals, or clinical samples.

### Method details

#### Overview of the analysis pipeline

This study builds directly on pre-trained latent encoders developed in our earlier work,^30^ where neural networks were trained to learn shared representations across heterogeneous electrochemical energy storage materials datasets using shared physicochemical descriptors, missingness masks, and domain-aware regularization. The latent encoder was optimized by backpropagation in a target-free setting, with electrochemical targets reserved exclusively for *post hoc* probing, attribution, and transfer analysis. In the present work, these pre-trained encoders are treated as fixed and are not retrained. All analyses, therefore, constitute a read-only audit of representation geometry, transferability, and deployment reliability, designed to interrogate how and where model predictions succeed or fail under domain shift.

The analysis pipeline proceeds in four stages. First, electrochemical energy storage materials data are embedded into a shared latent space using the pre-trained encoder from ref.^30^ Second, the geometry of this latent space is quantified through global distance, local density, and neighborhood structure, providing interpretable proxies for extrapolation and representational support. Third, transferability is evaluated *via* k-nearest-neighbor (kNN) relationships, enabling domain-pair-specific diagnostics of transferability and reliability that extend beyond aggregate performance metrics. Finally, latent geometry is integrated with epistemic uncertainty estimates to construct uncertainty-aware safety maps and deployment criteria, including rejection rules that identify extrapolative regimes where predictions should not be trusted.

A conceptual schematic summarizing the pipeline from the pre-trained encoder to the latent space, kNN graph, and reliability diagnostics is shown in Figure 4, highlighting how representation geometry underpins transferability and deployment safety without modifying the underlying model.

#### Data sources and domains

The analysis uses datasets of porous energy storage materials curated for our earlier work,^30^ and further feature engineered. These datasets show textural and electrochemical characterization features with ionic liquid 1-Ethyl-3-methylimidazolium tetrafluoroborate – (EMIMBF_4_) as a common electrolyte. Each sample is associated with a domain label indicating its origin:

• carbon_exp: experimentally measured porous carbons were obtained from ref.^37^^,^^38^^,^^39^^,^^40^^,^^41^^,^^42^^,^^43^^,^^44^^,^^45^^,^^46^
• mof_exp: experimentally measured metal–organic frameworks^47^
• mof_sim: simulated metal–organic frameworks^48^

In this work, additional datasets were compiled for inference-only, out-of-distribution (OOD) analysis.

• extra_carbon_exp: extra experimentally measured porous carbon datasets from ref.^49^^,^^50^
• extra_CTF_exp: extra experimentally measured covalent triazine-based frameworks (CTF) from ref.^9^

Input features are stored in a fixed-order numerical array $X\in R^{N\times d}$, where *N* is the number of samples and *d* is the feature dimensionality. Domain labels are provided separately and treated as categorical metadata.

Target properties (gravimetric - *C*_*g*_ and volumetric capacitance - *C*_*v*_) were available for the datasets analyzed in this study and were used to perform target-dependent nearest-neighbor target-property discrepancy and uncertainty analyses. For completeness, our pipeline is designed to fall back to geometry-only diagnostics when target labels are unavailable, although this mode is not used in the present work. Table 1 summarizes the dataset composition used for the reliability analysis reported herein.

#### Latent representation model

The reliability audit was performed using a fixed pre-trained feedforward neural network encoder generated during an earlier stage of the representation-learning workflow described in ref.^30^ The encoder audited in the present work corresponds to a specific archived representation artifact and is treated as a frozen deployment object. The present study does not use this encoder as a claim of architectural optimality, nor does it retrain, fine-tune, or update the representation. Instead, the encoder is used to evaluate whether latent geometry, local density, epistemic uncertainty, and nearest-neighbor target-property discrepancy can identify reliable, cautionary, and unsupported regimes under domain shift. Subsequent revisions or alternative encoders associated with ref.^30^ do not affect the interpretation of the present analysis, which is explicitly tied to the archived fixed encoder and preprocessing artifacts used here.

The encoder maps input feature vectors into a shared latent representation space.^52^ For a sample *i* with input feature vector x_*i*_, the latent embedding $z_{i}\in R^{k}$ is defined by Equation 1 as:

(Equation 1) $$z_{i}=f_{\theta }\left(x_{i}\right)$$

where *f*_*θ*_ denotes the fixed pre-trained encoder, and $z_{i}\in R^{k}$ is the corresponding latent embedding. The encoder architecture consists of two fully connected layers with ReLU activations and dropout. All encoder weights were frozen during the present analysis.

Latent embeddings were computed in evaluation mode for deterministic geometry analyses. Dropout layers were activated only during Monte Carlo dropout calculations, where repeated stochastic forward passes were used to estimate latent epistemic uncertainty. Thus, the present study audits the reliability structure of one fixed learned representation rather than benchmarking alternative encoder architectures or claiming that this encoder is universally optimal. This design separates the representation-learning step from the deployment-audit question addressed here: whether latent geometry, local representational support, epistemic uncertainty, and nearest-neighbor target-property discrepancy can identify interpolation-like, cautionary, and high-risk extrapolative regimes under domain shift.

#### Latent geometry metrics

##### Latent distance and density

For each sample, local latent geometry is characterized using k-nearest neighbors (kNN) in latent space. Euclidean distance is used throughout.

The latent distance, *d*, for sample *i* is defined as the mean distance to its *K* nearest neighbors (Equation 2):

(Equation 2) $$d_{i}=\frac{1}{K}\sum_{j\in N_{K}\left(i\right)}∥z_{i}-z_{j}{∥}_{2}$$

A latent density proxy (*ρ*) is then defined as the inverse of this distance (Equation 3):

(Equation 3) $$\rho_{i}=\frac{1}{d_{i}+\varepsilon }$$

where *ε* is a small constant to prevent division by zero.

These quantities serve as indicators of data support: samples with high density (low *d*_*i*_) lie in well-covered regions of representation space, while low-density samples indicate sparse or extrapolative regions.

#### kNN graph construction and domain mixing

To analyze cross-domain relationships, a directed k-nearest neighbor (kNN) graph^53^ was constructed in latent space. For each query sample *i*, directed edges were created to its *K* nearest neighbors *j*. Each edge was annotated with:

• latent distance, ∥z_*i*_-z_*j*_∥_2_
• query domain, *D*_*i*_
• neighbor domain, *D*_*j*_
• domain pair, *D*_*i*_→*D*_*j*_

This graph enables quantification of domain mixing, asymmetry in transfer directions, and domain-pair-specific reliability. Different *k* values were used for different diagnostic purposes. Local latent distance and latent density were computed using *k* = 10 nearest neighbors, because these quantities estimate local representational support and benefit from moderate smoothing against individual outliers. In contrast, the directed domain-pair transfer-edge analysis used *k* = 5 nearest-neighbor edges to preserve highly local query–neighbor relationships and avoid diluting domain-pair structure with more distant samples. To assess robustness, we repeated the transfer-envelope analysis using a relaxed setting with *k* = 10 transfer edges, 6 latent-distance bins, a minimum of 8 pairs per bin, and a minimum of 15 pairs per domain pair. The resulting sensitivity analysis is reported in Figure S1.

#### Nearest-neighbor target-property discrepancy

When target properties *y* are available, we compute an edgewise nearest-neighbor target-property difference between each query sample and its latent-space neighbor, as shown in Equation 4:

(Equation 4) $$\Delta_{ij}=|y_{i}-y_{j}|$$

where *i* is the query sample and *j* is a latent neighbor. This quantity is not intended to replace supervised prediction error. Instead, it measures local smoothness of the learned representation: if nearby samples in latent space have very different target values, then the representation is unreliable for transfer in that region. We therefore use Δ_*ij*_ as a local transfer-discrepancy proxy for auditing representation reliability under domain shift. Throughout this work, nearest-neighbor target-property discrepancy is interpreted as a local representation-smoothness diagnostic rather than a direct supervised prediction error.

#### Binned error envelopes and reliability boundaries

To quantify how nearest-neighbor target-property discrepancy scales with latent distance, nearest-neighbor target-property discrepancies are binned along the latent distance axis. For each bin, the following statistics are computed:

• median error
• interquartile range (25th–75th percentiles)
• 90th percentile error

These statistics define error envelopes that characterize expected and worst-case behavior as a function of representation distance. Envelopes are computed globally and separately for each domain pair, provided that sufficient data are available. Bootstrap resampling is used to estimate confidence intervals where applicable.

#### Epistemic uncertainty estimation

Epistemic uncertainty is estimated using Monte Carlo dropout applied to the latent encoder.^27^ During inference, dropout layers are activated and the encoder is evaluated multiple times for each input. This is represented by Equation 5 as:

(Equation 5) $$z_{i}^{\left(s\right)}=f_{\theta }^{\left(s\right)}\left(x_{i}\right),s=1,\ldots ,S$$

The latent epistemic uncertainty (*u*) for sample *i* is, therefore, defined by Equation 6 as:

(Equation 6) $$u_{i}=\frac{1}{k}\sum_{m=1}^{k}\sigma_{s}\left(z_{i,m}^{\left(s\right)}\right)$$

where *k* is the latent dimensionality and σ_*s*_ denotes the standard deviation across Monte Carlo samples. This uncertainty captures model sensitivity to perturbations in network parameters and reflects lack of epistemic support in representation space.

#### Safety maps and traffic-light classification

Latent distance and epistemic uncertainty are combined into a two-dimensional deployment safety map. Thresholds are defined using the 90th percentile of the training distribution as an empirical measure (Equation 7):

(Equation 7) $$d_{\text{thr}}=Q_{0.9}\left(d_{i}\right),u_{\text{thr}}=Q_{0.9}\left(u_{i}\right)$$

Each sample is assigned a qualitative safety label:

• Safe: *d*_*i*_ ≤ *d*_thr_ and *u*_*i*_ ≤ *u*_thr_
• Caution: one threshold exceeded
• Unsafe: both thresholds exceeded

These regions provide interpretable guidance for deployment decisions. Safety thresholds were defined using the internal training-domain distribution. These thresholds were first visualized for the training-domain datasets in Figure 1E and then applied unchanged to external inference-only datasets in the deployment/OOD analysis shown in Figure 3B.

#### Inference-only and out-of-distribution evaluation

Additional experimental datasets (e.g., new experimentally measured porous carbons and CTFs) were introduced solely at inference time. These samples were:

1. Embedded using the same pre-trained encoder
2. Evaluated for latent distance to the training distribution
3. Assigned epistemic uncertainty *via* MC dropout

No target values were required. These analyses enabled OOD detection and deployment risk assessment for emerging material classes. Out-of-distribution behavior was interpreted using latent-space distance, local density, and uncertainty diagnostics, consistent with prior work on OOD behavior in learned representations and uncertainty-controlled chemical discovery.^32^

Additional methodological details, sensitivity analyses, and representative target-resolved reliability cases are provided in the Supplementary Information.

### Quantification and statistical analysis

All quantitative analyses used fixed embeddings generated from the archived pre-trained encoder and preprocessing artifacts. No model retraining, fine-tuning, or hyperparameter optimization was performed. Local latent distance and density were computed from k-nearest neighbors. Directed domain-pair transfer relationships used k = 5 nearest-neighbor edges, whereas local distance and density estimates used k = 10. Sensitivity analyses repeated the transfer-edge analysis using k = 10, 6 distance bins, at least 8 pairs per bin, and at least 15 pairs per domain pair.

For labeled samples, nearest-neighbor target-property discrepancy was quantified as the absolute difference in *C*_*g*_ or *C*_*v*_ between a query sample and its latent neighbor. Distance-binned envelopes report medians, interquartile ranges, and 90th percentiles within each bin. Epistemic uncertainty was estimated using Monte Carlo dropout with the frozen encoder. A neighborhood-based target-property variability proxy was also used as a complementary diagnostic where labels were available.

Safety maps used empirical 90th-percentile thresholds from the internal training-domain distribution for latent distance and epistemic uncertainty. External datasets were evaluated using the same thresholds without recalibration.

Internal leave-one-out latent-neighborhood validation was performed for samples with available *C*_*g*_ and *C*_*v*_ labels by estimating each target from its nearest latent neighbors after excluding the query sample.

Bootstrap resampling estimated uncertainty in selected summary statistics. No formal hypothesis testing was used because the study is a representation-reliability audit rather than a test of group-level statistical significance. All analyses were implemented in Python 3.13.3 on Windows 11. The main computational environment used NumPy 2.4.4, pandas 3.0.2, SciPy 1.17.1, scikit-learn 1.8.0, joblib 1.5.3, PyTorch 2.11.0, UMAP-learn 0.5.12, Plotly 6.7.0, openpyxl 3.1.5, nbformat 5.10.4, and Kaleido 1.3.0. Random seeds were fixed where applicable to improve reproducibility, and analysis parameters, software versions, selected artifacts, and file paths were recorded in the accompanying reproducibility manifest.

## Supplemental information

Document S1. Figures S1–S4

Table S1. Representative sample-level reliability regimes identified from latent-neighborhood diagnostics

## Footnotes

Supplemental information can be found online at https://doi.org/10.1016/j.isci.2026.117160.
