# Supplementary material for: Reliable machine learning deployment across domains in electrochemical energy storage materials
Source: iScience. 2026 Aug 14;29(9):117160. doi: 10.1016/j.isci.2026.117160 (PMC13503088; doi:10.1016/j.isci.2026.117160)
Supplement: Supplementary file 1 [file mmc1.pdf]

iScience, Volume *29*

Supplemental information

Reliable machine learning deployment across domains in electrochemical energy storage materials

Henry R.N.B. Enninful and Muhammad Abdullah Khan

Table of Contents

S1. Additional Methods 2

S1.1 Domain-pair target-property discrepancy analysis 2

S1.2 Local target-property variability proxy 2

S1.3 Exploratory calibration analysis 2

S1.4 Reproducibility and implementation 3

S1.5 Data and code availability 3

S2. Extended Results 4

S2.1 Sensitivity of domain-pair target-property discrepancy envelopes to relaxed neighborhood thresholds 4

S2.2 Latent geometry governs local target-property smoothness and uncertainty 6

S2.3 Internal validation against leave-one-out latent-neighborhood prediction error.. 8

S2.4 Representative reliability cases in distance–uncertainty space 9

S1. Additional Methods

S1.1 Domain-pair target-property discrepancy analysis

To quantify how local target-property smoothness changes across materials domains, we constructed domain-pair discrepancy tables for each target property. For a given target and ordered domain pair, nearest-neighbor target-property discrepancies were computed between each query sample and its latent nearest neighbors. Samples were stratified into near- and far-neighbor regimes based on the first and third quartiles of the latent-distance distribution, respectively. The median absolute nearest-neighbor target-property discrepancy in these regimes provides a compact summary of how local structure–property consistency changes with latent-neighbor distance and enables direct comparison across domain pairs.

Nearest-neighbor target-property discrepancy is interpreted throughout as a local representation-smoothness diagnostic rather than as direct supervised prediction error.

S1.2 Local target-property variability proxy

As a complementary target-dependent diagnostic, we computed a local target-property variability proxy based on the dispersion of target values within latent neighborhoods. For each sample i, the proxy is defined as the standard deviation of the target values of its k-nearest neighbors in latent space:

$$\sigma_i^{\mathrm{local}} = \mathrm{Std}\big(y_j \mid j \in \mathcal{N}_k(i)\big)$$

where $\mathcal{N}_k(i)$ denotes the set of k-nearest neighbors of sample i, and y_j is the corresponding target value of neighbor j. This metric estimates local target-space heterogeneity around each sample and is used to assess whether latent neighborhoods preserve smooth target-property behavior.

This quantity is not a model-derived epistemic uncertainty. Instead, it is a neighborhood-based target-variability diagnostic that complements the Monte Carlo dropout uncertainty used in the main analysis.

S1.3 Exploratory calibration analysis

As an exploratory extension, we tested whether latent distance and epistemic uncertainty could be converted into calibrated probability-of-safety scores for selective deployment. A binary safety label was defined using nearest-neighbor target-property discrepancy as a local representation-smoothness proxy, and logistic calibration was evaluated using latent distance and epistemic uncertainty as input descriptors.

These exploratory analyses were not used for quantitative interpretation in the main text because the available labeled data did not provide sufficiently balanced coverage of intermediate-risk regimes. In particular, the derived safety labels were imbalanced, and sparsely populated domain regions led to unstable calibration. Therefore, calibrated probability-of-safety scores and risk–coverage curves are not reported as formal results.

The main conclusions instead rely on directly interpretable, non-parametric diagnostics: latent distance, local density, epistemic uncertainty, and nearest-neighbor target-property discrepancy. This exploratory result does not affect the conclusions of the study, because the proposed reliability audit does not depend on probabilistic calibration.

S1.4 Reproducibility and implementation

All analyses were implemented in Python using PyTorch, NumPy, pandas, scikit-learn, SciPy, and Plotly. Random seeds were fixed where applicable to improve reproducibility. k-nearest-neighbor construction, bootstrapping, Monte Carlo dropout sampling, relaxed sensitivity analysis, representative-case selection, and figure generation are specified in the accompanying analysis script.

S1.5 Data and code availability

All analyses operate on fixed pretrained artifacts generated during the earlier representation-learning workflow, including latent encoders, feature scalers, and target data arrays. No model training, fine-tuning, or hyperparameter optimization was performed in the present study. File paths, selected artifacts, software versions, and analysis parameters are recorded in the reproducibility manifest generated by the analysis script.

Because the present work audits one frozen latent representation, the reported reliability boundaries should be interpreted as specific to that learned representation rather than as universal boundaries for all possible encoder architectures.

S2. Extended Results

S2.1 Sensitivity of domain-pair target-property discrepancy envelopes to relaxed neighborhood thresholds

To assess the robustness of the domain-pair nearest-neighbor target-property discrepancy envelopes reported in the main text, we repeated the analysis using relaxed neighborhood and binning parameters. Specifically, the k-nearest-neighbor graph was rebuilt using a larger neighborhood size ($k = 10$), nearest-neighbor target-property discrepancies were aggregated into fewer latent-distance bins (6 bins), and the minimum number of sample pairs required per bin and per domain pair was reduced to eight pairs per bin and fifteen pairs per domain pair, respectively. These settings increase coverage for sparsely sampled domain pairs while preserving the same geometric definitions of latent distance and nearest-neighbor target-property discrepancy.

Figure S1 shows the resulting relaxed discrepancy envelopes for both gravimetric and volumetric capacitance targets. Across both properties, the qualitative trends observed in the main analysis are preserved. Simulated MOF–simulated MOF neighbor pairs continue to exhibit comparatively low median discrepancy and relatively narrow interquartile ranges across the retained latent-distance bins, indicating stable local structure–property consistency within the learned representation. In contrast, carbon–carbon and experimental MOF–experimental MOF domain pairs show stronger increases in discrepancy and/or envelope width as latent-neighbor distance increases, consistent with reduced local target-property smoothness in more distant regions of latent space.

Although the relaxed thresholds introduce increased variability in sparsely populated bins, no domain pair shows a systematic recovery of low discrepancy at large latent-neighbor distances. This supports the conclusion that the observed distance-dependent degradation in local target-property smoothness and the relative ordering of domain-pair behavior are not artifacts of conservative filtering choices. As in the main analysis, nearest-neighbor target-property discrepancy is interpreted as a local representation-smoothness diagnostic rather than a direct supervised prediction error.

a

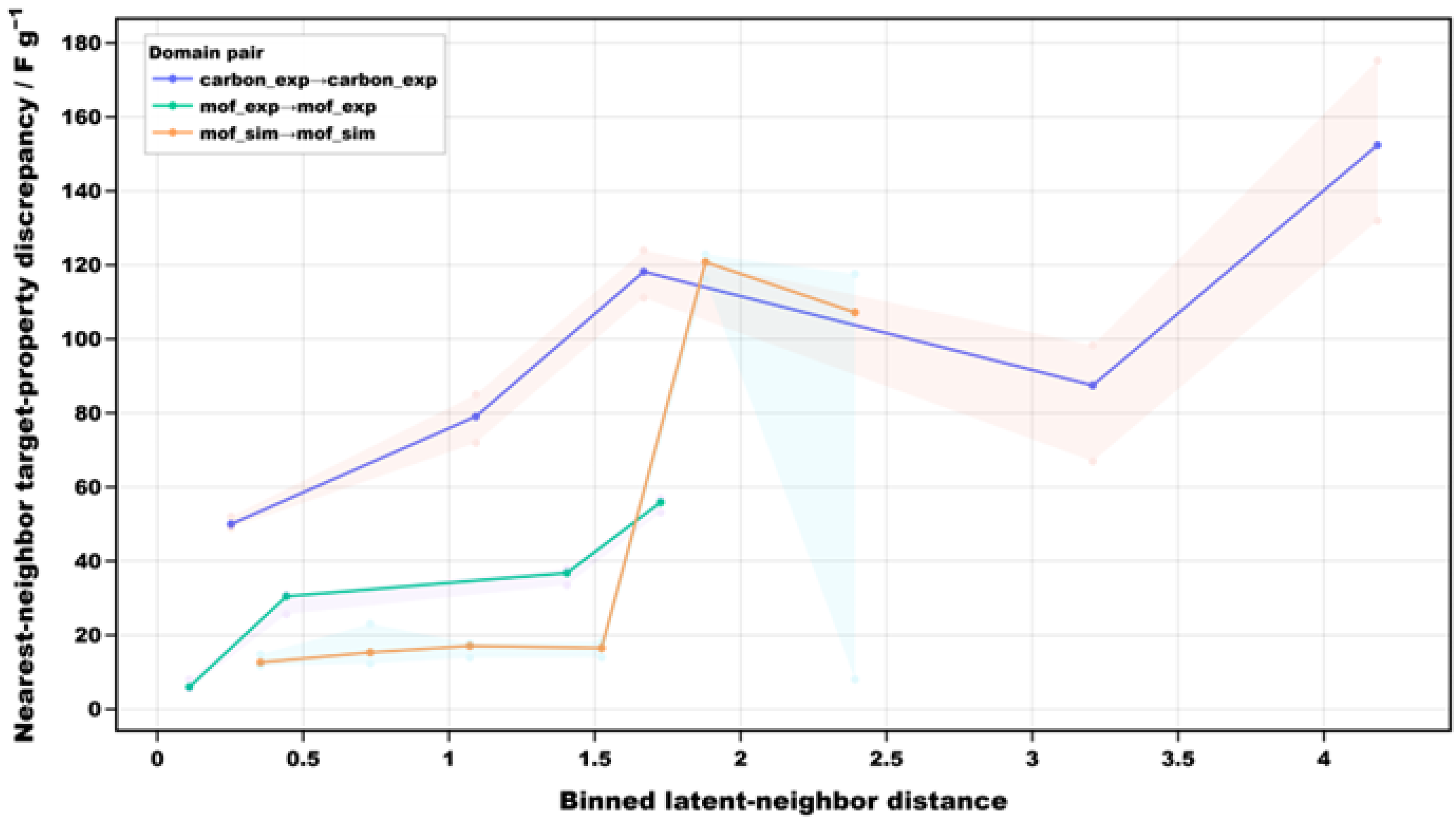

b

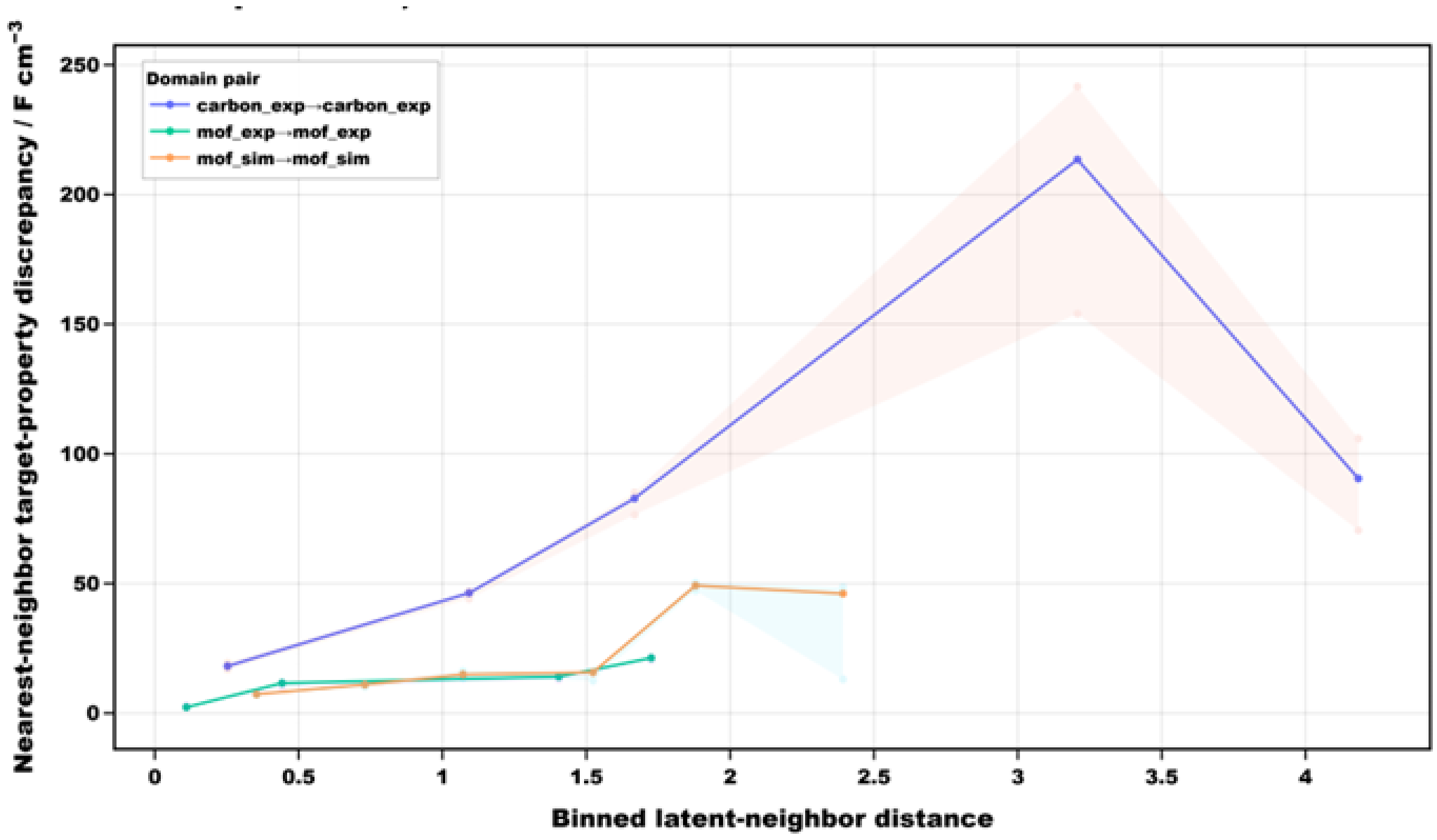

Figure S1. Sensitivity of distance-binned nearest-neighbor target-property discrepancy envelopes to relaxed analysis thresholds. Distance-binned median nearest-neighbor target-property discrepancy and interquartile ranges computed using relaxed neighborhood and binning parameters (k = 10 neighbors, 6 distance bins, minimum 8 pairs per bin, and minimum 15 pairs per domain pair). Results are shown separately for gravimetric (a) and volumetric capacitance (b) targets. The qualitative dependence of nearest-neighbor target-property discrepancy on latent distance and the relative ordering of materials domains remain unchanged, confirming the robustness of the main-text conclusions to reasonable variations in analysis hyperparameters.

S2.2 Latent geometry governs local target-property smoothness and uncertainty

To further examine how latent-space geometry controls deployment risk, we analyzed the relationship between local density, latent distance, nearest-neighbor target-property discrepancy, and neighborhood-based target variability. Figure S2 summarizes these supplementary diagnostics for gravimetric and volumetric capacitance.

Figure S2a shows that large nearest-neighbor target-property discrepancies are concentrated primarily in low-density regions of latent space. At higher local density, the discrepancy distribution narrows substantially for both gravimetric and volumetric capacitance, indicating that densely sampled latent neighborhoods are more likely to preserve local target-property similarity. This supports the interpretation of latent density as a measure of local representational support.

Figure S2b compares a neighborhood-based target-variability proxy with latent distance. The local target-property variability generally increases in more distant regions of latent space, although the trend is not strictly monotonic across all domains. Carbon samples show broader variability at larger latent distances, whereas simulated MOFs remain comparatively more compact over much of the sampled range. This indicates that latent distance captures extrapolative displacement but should be interpreted jointly with local density and domain-specific neighborhood structure.

Figure S2c shows that local target-property variability is reduced in denser latent neighborhoods, particularly for simulated MOFs and portions of the carbon domain. Conversely, low-density regions contain several high-variability samples, especially for carbon materials. Together, these trends support the central conclusion that local representational support, rather than domain identity alone, governs the smoothness of target-property behavior in the learned representation.

Importantly, the quantities plotted in Figure S2 are diagnostic measures of local representation quality. Nearest-neighbor target-property discrepancy and local target-property variability should therefore be interpreted as representation-smoothness and deployment-risk indicators, not as direct substitutes for independently measured supervised prediction error.

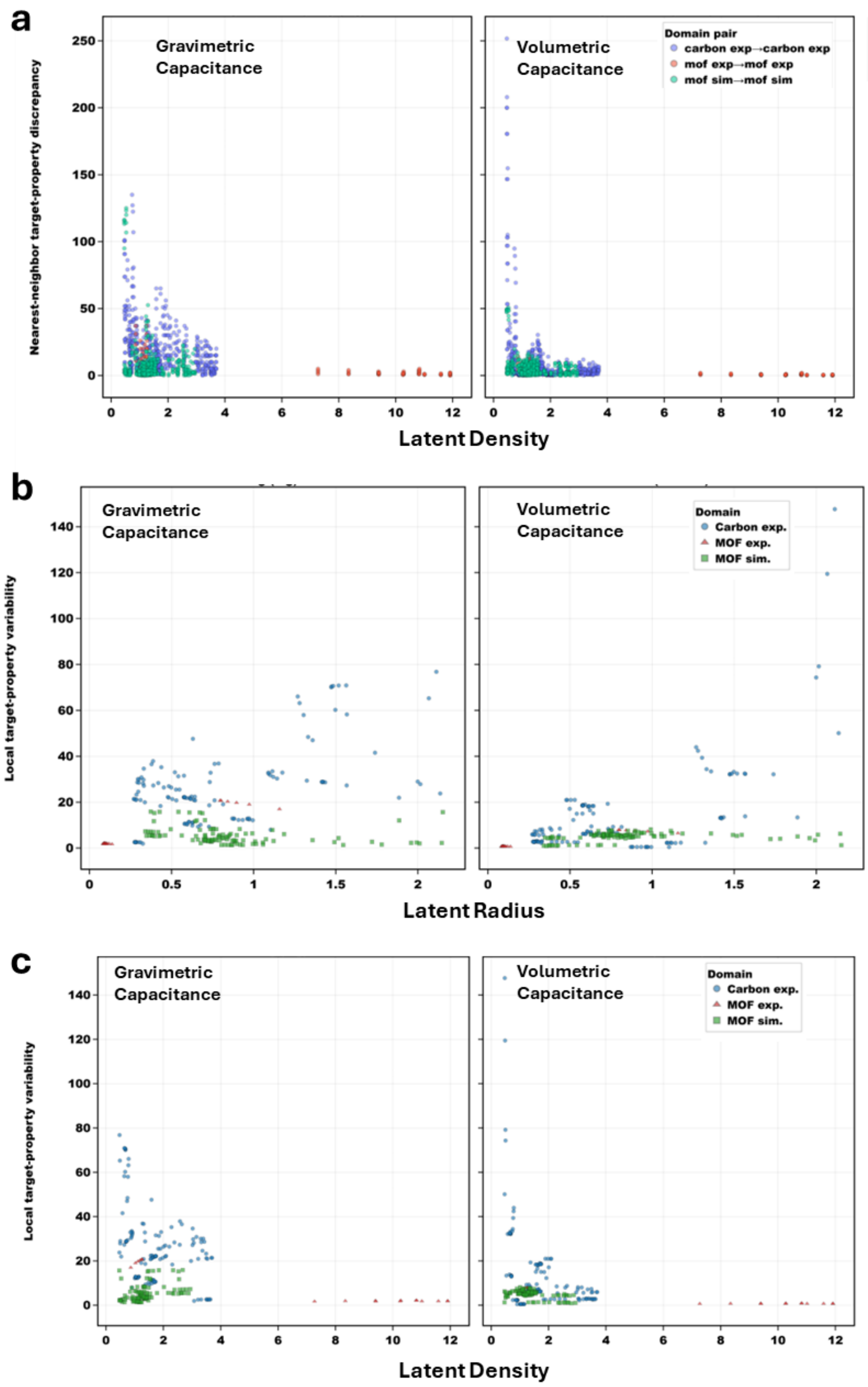

Figure S2. Latent geometry governs local target-property smoothness and uncertainty. (a) Absolute nearest-neighbor target-property discrepancy as a function of latent density for gravimetric and volumetric capacitance. Large discrepancies are concentrated primarily in low-density regions, indicating reduced local target-property smoothness where representational support is weak. (b) Neighborhood-based target-property variability as a function of latent distance. Local variability generally increases in more distant regions of latent space, although the trend is domain-dependent and not strictly monotonic. (c) Neighborhood-based target-property variability as a function of latent density, showing reduced variability in denser latent neighborhoods. These diagnostics support the use of latent density, latent distance, and local target-property variability as complementary indicators of deployment risk rather than direct measures of supervised prediction error.

S2.3 Internal validation against leave-one-out latent-neighborhood prediction error

To assess whether the proposed reliability indicators are associated with predictive degradation, we performed an internal leave-one-out latent-neighborhood validation for samples with available gravimetric capacitance, C_g, and volumetric capacitance, C_v, labels. For each labeled sample, the target value was estimated from its nearest latent neighbors after excluding the query sample. The resulting absolute prediction error was then compared with latent distance, local density, epistemic uncertainty, and nearest-neighbor target-property discrepancy.

This validation is intended as a supporting analysis rather than a replacement for prospective external testing. Nearest-neighbor target-property discrepancy is therefore interpreted as a local representation-smoothness diagnostic, not as direct prediction error. Directional associations between the leave-one-out prediction error and the reliability indicators support their use as conservative warning signals for deployment risk.

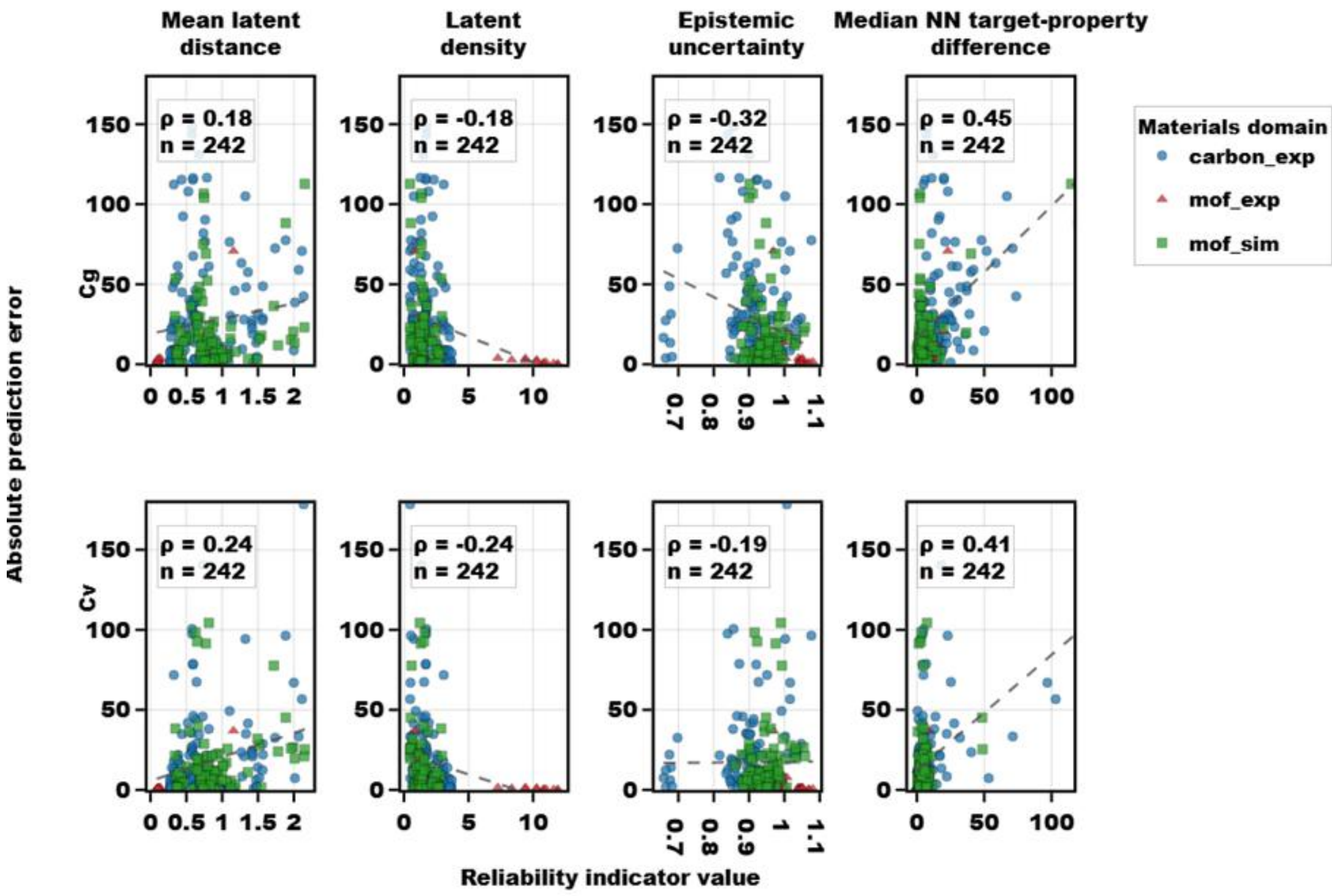

Figure S3. Internal validation of reliability indicators against leave-one-out latent-neighborhood prediction error. Absolute prediction error is compared with mean latent distance, latent density, epistemic uncertainty, and median nearest-neighbor target-property difference for gravimetric capacitance, C_g, and volumetric capacitance, C_v. Rows correspond to C_g and C_v, respectively. Dashed lines indicate linear trend guides, and Spearman correlation coefficients are shown in each panel. These indicators are interpreted as conservative deployment-risk diagnostics rather than direct substitutes for external supervised prediction-error estimates.

S2.4 Representative reliability cases in distance–uncertainty space

To visualize the sample-level regimes reported in Table S1, we mapped the representative cases onto the latent distance–epistemic uncertainty safety space. Figure S4 shows the selected cases for gravimetric capacitance, C_g, and volumetric capacitance, C_v. The dashed vertical and horizontal lines denote the internal thresholds used to define interpolation-like, cautionary, and high-risk regions based on latent-neighbor distance and epistemic uncertainty.

Case A corresponds to a reliable interpolation-like regime, where the selected sample lies within a well-supported region of latent space and exhibits low nearest-neighbor target-property discrepancy. Case B illustrates a silent-failure-risk regime, where epistemic uncertainty is not necessarily extreme, but local target-property discrepancy is high. Case C represents a sparse extrapolative regime, where large latent distance and weak local support indicate elevated deployment risk. These examples show that latent distance, epistemic uncertainty, local density, and nearest-neighbor target-property discrepancy must be interpreted jointly rather than in isolation.

Because the selected cases are target-resolved, the representative samples may differ between C_g and C_v. The figure is therefore intended as a visual companion to Table S1 rather than as an independent prediction-error analysis. Nearest-neighbor target-property discrepancy is interpreted here as a local representation-smoothness diagnostic, not as a direct substitute for supervised prediction error.

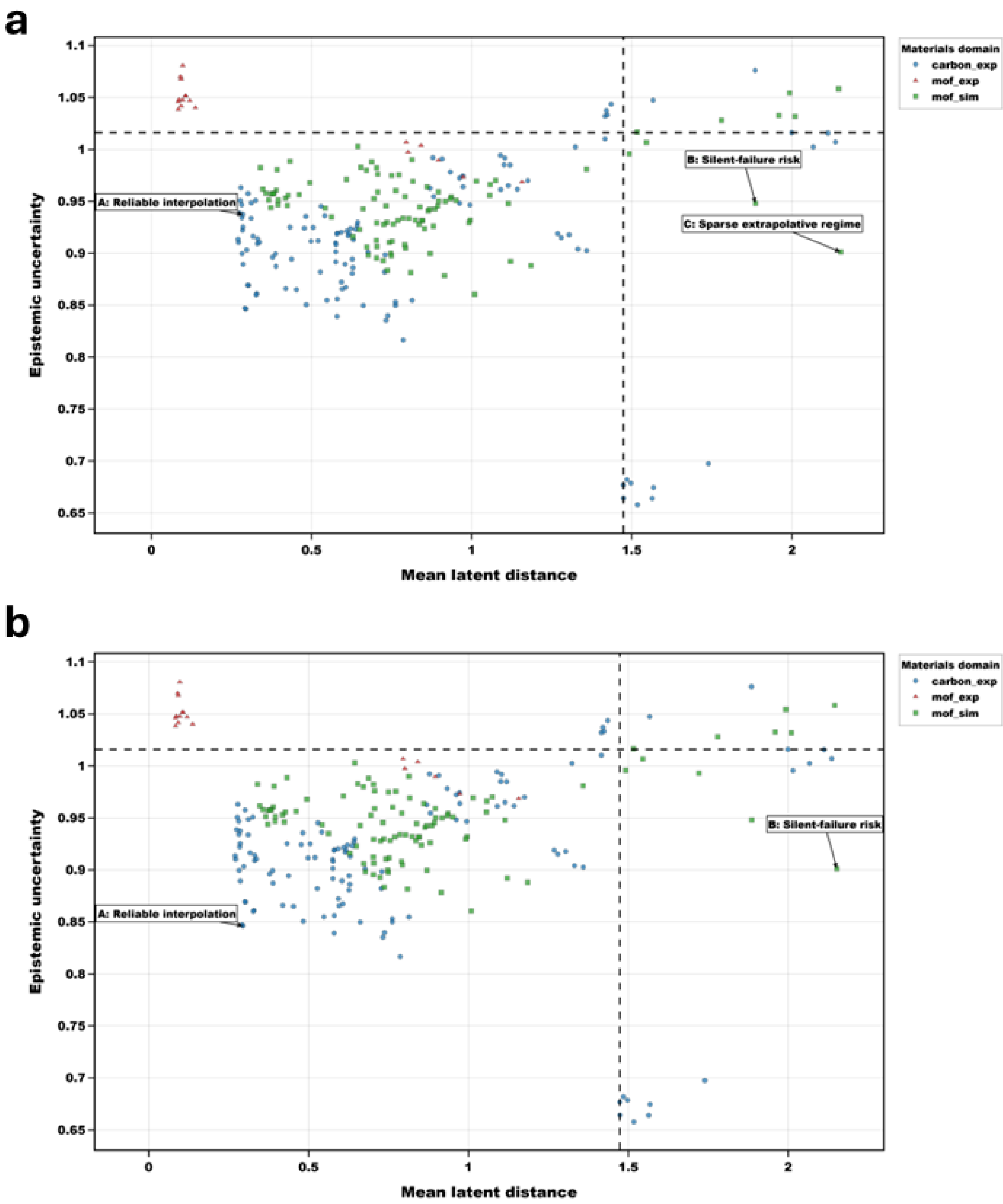

Figure S4. Representative reliability cases in distance–uncertainty space. Representative cases selected for Table S1 are mapped onto the latent distance–epistemic uncertainty safety space for (a) gravimetric capacitance, C_g, and (b) volumetric capacitance, C_v. Dashed lines indicate the internal latent-distance and epistemic-uncertainty thresholds used to define interpolation-like, cautionary, and high-risk deployment regimes. Case A denotes a reliable interpolation-like regime, Case B denotes silent-failure risk, and Case C denotes a sparse extrapolative regime. These examples illustrate that local target-property discrepancy, latent distance, and uncertainty provide complementary deployment-risk diagnostics.
